# Do Polish Consumers Take Proper Care of Hygiene while Shopping and Preparing Meals at Home in the Context of Wasting Food?

**DOI:** 10.3390/ijerph17062074

**Published:** 2020-03-20

**Authors:** Marzena Tomaszewska, Beata Bilska, Danuta Kołożyn-Krajewska

**Affiliations:** Warsaw University of Life Sciences – SGGW, Institute of Human Nutrition Sciences, Department of Food Gastronomy and Food Hygiene, 159C Nowoursynowska St., 02-776 Warsaw, Poland; beata_bilska@sggw.pl (B.B.); danuta_kolozyn_krajewska@sggw.pl (D.K.-K.)

**Keywords:** consumers, food safety, food safety knowledge, food safety practice, food waste, food spoilage, cluster analysis

## Abstract

The objective of this paper is to evaluate the knowledge and practices of Polish consumers in terms of broadly defined hygiene on food preparation at home. The consequence of improper food handling may be a faster rate of food spoilage. A specially designed questionnaire was used. The study was conducted on a nationwide, random, and representative group of 1115 adult respondents. Segmentation (cluster analysis) of respondents differing in their practice and knowledge of meal preparation and personal hygiene was carried out. Several areas were diagnosed in which the respondents’ knowledge and practice were insufficient, such as storage of food products, inappropriate conduct of the thawing process, and heating of dishes. It was found that the best practice and knowledge of the issues discussed were characteristic of unemployed women over 35 years of age (cluster D). They offen gave answers that were significantly different (*p* < 0.05) from those given by the other clusters. The most limited knowledge and the worst practices were characteristic of mainly men with elementary and secondary education who are a part of the labor force (cluster E and A). The segmentation provided valuable information which indicates that educational programs on food safety need to be further strengthened.

## 1. Introduction 

The importance of food safety is recognised worldwide, especially in developed countries. However, despite growing knowledge in both developed and developing countries, foodborne diseases still persist. 

The most common symptom resulting from consumption of contaminated food is gastrointestinal discomfort, including diarrhea, which occurs in 550 million people worldwide every year and causes 230,000 deaths [1].

Sanlier [2] notes that foodborne diseases are common in both developing and developed countries, although the causes of foodborne diseases can be slightly different. In less-developed countries, the causes can include unhygienic conditions during preparation and consumption of foods, contaminated water, unfavorable weather conditions (drought), inappropriate food storage conditions, and pesticide residue. In developed countries, the causes can include inadequate food storage and preparation conditions and the demand for cheap food. In industrialized countries, foodborne illnesses are usually characterized by mild symptoms. However, they represent a major challenge to public health due to a significant financial burden and possible serious health consequences [3,4].

For the year 2017, a total of 5079 foodborne and waterborne disease outbreaks were reported by 27 EU countries (372 less than in 2016), which corresponds to 97.7 outbreaks per week, on average, at the EU level. France reported the largest number of outbreaks and accounted for more than one-quarter of all outbreaks reported in 2017 in the EU. Outbreaks reported by another seven countries (Belgium, Germany, the Netherlands, Poland, Slovakia, Spain, and Sweden) accounted altogether for more than 60% of total reported food and waterborne disease outbreaks. In the EU, in the 43,400 involved cases (13,519 less than in 2016), 4541 hospitalizations (119 more than in 2016) and 33 deaths (one more than in 2016) were reported [5]. Jevšnik et al. [6] pointed out that consumers are an important link in the food safety chain when considering a “from farm to table” approach, because a large proportion of foodborne illness originates in the home [4]. In the 27 EU countries, “household” was the most frequently reported place of exposure to contaminated foods and one in three outbreaks occurred in this setting [5].

For this reason, many studies have focused on food safety knowledge and behavior among various groups of consumers: adults [7,8], families with young children [9,10,11], elderly persons [12], and young adults and students [2,13,14,15]. On the basis of these surveys, it is possible to indicate areas of conduct and knowledge that should be given special emphasis in educational activities, taking into account a specific group of respondents. In order to minimize the risk of foodborne diseases, it is necessary to constantly educate consumers regardless of their age, place of residence, and level of education. Consumers need to know which behaviors are most likely to result in illness in order to make decision about their food handling and consumption behaviors [12]. Nowadays, all kinds of educational activities in the field of food safety are becoming particularly important due to the increasingly widespread phenomenon of antibiotic resistance when the foodborne illness is caused by bacteria. It is estimated that every year in the EU countries alone, approximately 25,000 patients die from bacterial infections demonstrating resistance to antibiotics [16]. 

This topic is important for more than food safety reasons and has another specific dimension related to food security, which is increasingly being discussed in the context of a sustainable system. It is estimated that more than 1/3 of the total food produced is wasted, which is a serious problem that generates negative social, environmental, economic, and ethical impacts. All links in the food chain are responsible for this disgraceful practice. However, the last of them, i.e., households, bear the greatest responsibility for food waste. In the European Union (EU-28, data for 2012) households were responsible for more than 50% of wasted food [17]. Food waste in households is the result of various closely related factors. Many researchers dealing with the phenomenon of food waste in households focus on sociodemographic factors such as the size of the household [18,19,20,21], the number of children in the household [22], the age of consumers [22,23], and the household income level [24]. They take into account aspects such as preparation for shopping [20,25,26], conduct of the shopping itself [27], the size of the packaging [28], and planning of meals at home [29,30,31]. Meanwhile, many influencing factors on food waste hide silently behind routine household activities (shopping, storing, cooking, and serving food) as conative attitude elements [32]. There are no studies that focus on aspects related to hygiene during shopping, storing of food, and preparation of meals in the context of food waste in households. As proven in the studies by Bilska et al. [33], one of the basic reasons for throwing food away in Polish households is its spoilage. Of course, food spoils because consumers make unreasonable, excessive purchases [34]. But the rate at which it spoils can also be influenced by the failure to comply with process conditions and individual operations, as well as hygiene standards, during processing. For example, abusive storage conditions result in microbial spoilage of foods and hence these products are not consumed [35]. Long exposure of perishable food at room temperature can promote growth of any pathogenic or spoilage bacteria present in the product. People in general are aware of the issue of food waste to some extent if asked, but habits that lead to an increased amount of unnecessary food waste tend to be unconscious in nature [36].

Therefore, the objective of this paper is to evaluate the knowledge and practices of Polish consumers in terms of broadly defined hygiene on meal preparation at home, including during shopping, storage, and preparation of food, and personal hygiene. Moreover, segmentation of Polish consumers was also carried out in order to identify groups characterized by similar food handling behaviors, with particular emphasis on food safety. The following questions were asked: (1) Is handling of food in households linked to sociodemographic characteristics such as gender, age, education, or labor force participation? (2) Can the observed consumer behavior potentially contribute to food waste?

## 2. Materials and methods

### 2.1. Date Collection

The study was conducted in February and March 2019 on a group of 1115 adult respondents who agreed to take part in the survey. The selection of the sample from the address survey of Central Statistical Office in Poland fulfilled the condition of representativeness of the general population for Polish residents older than 18 years in terms of gender, age, and the size of the place of residence. The survey was conducted in each of the sixteen provinces of Poland. After drawing the starting addresses, the so-called random route method was used to select the sample. The study was conducted using the Computer Assisted Personal Interview (CAPI) technique. 

Prior to the proper study, a pilot study was conducted with 30 participants. All the concerns/problems raised by the respondents were discussed and taken into account in the questionnaire. The interview, using a revised questionnaire, was conducted by trained pollsters. During the study, irregularities were found in the case of 23 interviews (i.e., too short time of interview or suggesting answers by the pollster). These data were removed from the database and interviews were conducted with other respondents. 

Table 1 shows the sociodemographic characteristics of the respondents. A comparable number of women and men took part in the proper study. The least numerous group by age was respondents aged 35–44. The percentage shares of other age groups were similar. People with secondary education were the largest group of respondents. Only fewer than one in five respondents declared higher education. A majority of the respondents declared a village as their place of residence (38.2%).

### 2.2. Questionnaire

**Figure 1:**
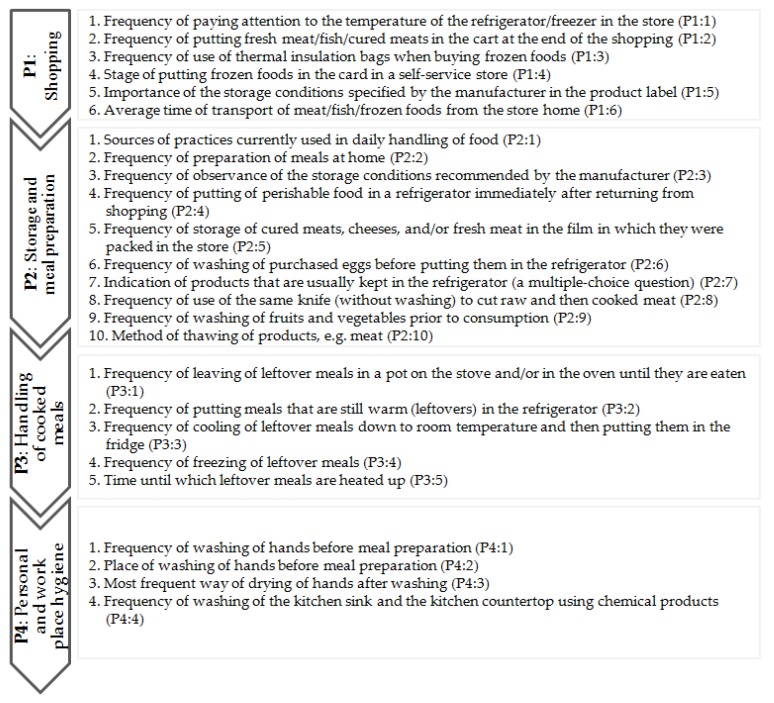
Arrangement and designation of issues covered by the consumer practice part of the questionnaire (P).

The questionnaire consisted of three parts. The first part contained 25 questions related to the handling of food by consumers when shopping (P1) and at home (P2 and P3), and to personal and workplace hygiene issues (P4) (Figure 1). The answers to the questions about the frequency of activities were based on a 5-point scale, with boundary terms “always” to “never” (**P1**: 1,2,3; **P2**: 2,3,4,5,6,8,9; **P3**: 1,2,3,4; **P4**: 1,4) and “definitely relevant” to “definitely irrelevant” (**P1**:5). For the other questions (**P1**: 4,6; **P2**: 1,7,10; **P3**: 5; **P4**: 2,3), multiple-choice answers were proposed.

**Figure 2:**
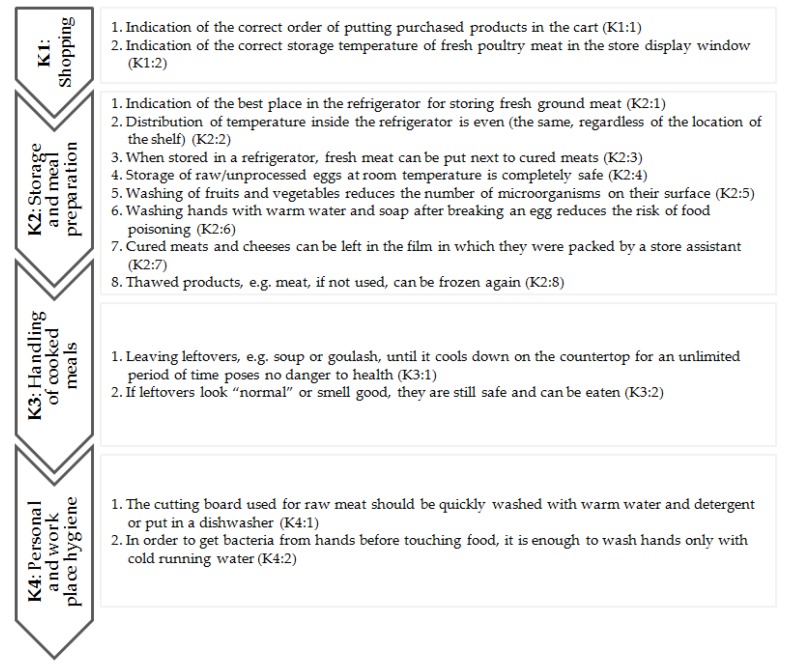
Arrangement and designation of issues covered by the consumer knowledge part of the questionnaire (K).

The second part of the questionnaire contained 14 questions related to the respondents’ knowledge of the issues raised in the first part of the questionnaire (Figure 2). For questions related to shopping (**K1**: 1,2) and one of the areas of storage and preparation of meals (**K2**: 1), multiple-choice answers were proposed. The answers to the remaining questions (from **K2**: 2 to **K4**: 2) were based on a 5-point Likert scale with boundary definitions “I strongly agree” to “I strongly disagree.”

Section 3 contained questions concerning the demographic and social affiliation of the respondents, i.e., their gender, age, education level, inhabitancy (place of origin).

### 2.3. Statistical Analysis

Taxonomic analysis was performed using the Statistica 12.1 software (StatSoft, Cracow, Poland). The purpose of the analysis was segmentation of respondents differing in their behavior and knowledge of meal preparation and personal hygiene. The multidimensional cluster analysis method was used for this purpose. The hierarchical Ward method was used to create clusters, using Euclidean distances [37]. Homogeneous clusters of respondents were identified on the basis of the average level of the arithmetic mean and the value of the fractional index. The clusters were determined on the basis of the distances of bonding from the bonding stages. On this basis, five clusters were identified. 

The cluster analysis was complemented by examining the significance of the differences between the average level of each element (consisting of a multidimensional criterion of cluster formation) in the identified clusters. The zero hypothesis of equal value of the mean/fraction index (calculated for each cluster) was verified by way of the Fisher–Snedecor F test and the post-hoc analysis was performed by way of the least significant difference (LSD) test. This enabled indication of homogeneous arithmetic mean groups. This verification was performed at the significance level of α = 0.05. 

## 3. Results

### 3.1. Behavior of Polish Consumers at Different Stages of Food Management in Terms of Food Safety

Based on the results of the study, it was found that Polish consumers sometimes act incorrectly at the various stages of food handling, from shopping to the handling of ready-made meals (Table 2). 

Only 1 in 10 respondents declared that in the shop they ‘always’ or ‘usually’ pay attention to the temperature of the fridge/freezer in which perishable products are placed (P1: 1). On the other hand, 1 in 5 respondents “always” or “usually” puts fresh meat, fish, and sausages in the cart at the end of shopping, after buying other products (P1: 2). Respondents indicated that when purchasing frozen food, they most often put it in the cart according to the arrangement of stands in a given store (P1: 4), and only less than 14% of them use heat-insulating bags to transport it (P1: 3). It was determined that the average time of transport of perishable products or frozen products from the store to the home for the vast majority of the respondents does not exceed 20 min (P1: 6).

The respondents participating in the survey indicated that they learned the food handling methods that they currently use in everyday life mainly from their parents and on their own, through life experience (P2: 1). 

More than half of the respondents declared regular preparation of meals at home (P2: 2). It was found that a comparable number of respondents, i.e., approximately 3/4 each, indicated the relevance of the storage conditions indicated by the manufacturer on the product label (P1: 5), their observance at home (P2:3), and placing perishable products in the refrigerator immediately after bringing them home (P2: 4). On the other hand, as many as 2/3 of the respondents declared storing cured meats, cheese, and fresh meat in the film in which it was packed in the shop (P2: 5), and 4 out of 10 declared washing eggs before placing them in the refrigerator (P2: 6). On the basis of answers given by the respondents, the vast majority (approximately 80%) declared proper storage conditions, i.e., at refrigeration temperature, for products such as fresh poultry and fish, yoghurts and buttermilk, cured meats, and fresh milk. On the other hand, only slightly more than 60% of them declared storing juices in the fridge after the package is opened (P2: 7). Nearly 70% of the respondents indicated inadequate thawing conditions for products, e.g., by immersion in warm water or by leaving them at room temperature (P2: 10).

It was found that almost half of the respondents indicated that a meal that has been prepared earlier is heated only until the meal reaches a temperature that allows for eating it immediately, i.e., it is warm but not too hot. The last part of the questionnaire concerning food handling shows that Poles make sure to wash their hands before they prepare a meal; they usually wash them in the kitchen sink (approximately 68%) and wipe them with a cloth intended for wiping hands in the kitchen or with disposable paper towels (28.2% and 26.2% of indications, respectively). 

### 3.2. Knowledge of Polish Consumers Concerning Food Management in Terms of Food Safety

It was found that Polish consumers have incomplete knowledge in areas such as the temperature at which fresh poultry meat should be stored in a refrigerated shop window, or the temperature distribution in the home refrigerator (Table 3). A significant majority of the respondents (approximately 60%) also indicated incorrect answers to questions K2: 4 and K2: 7, agreeing or declaring lack of knowledge of the statements, respectively, that “it is completely safe to store raw/ unprocessed eggs at room temperature” and “cured meats and cheese can be left in the film in which they were packed by the shop assistant.” Polish consumers were the most successful with statements concerning issues such as reduction of the number of microorganisms on the surface of fruits and vegetables by washing (K2: 5), the need to quickly wash small kitchen equipment (K4: 1), and the ban on re-freezing previously thawed products, e.g., meat (K2: 8). 

### 3.3. Sociodemographic Characteristics of Isolated Clusters

On the basis of the answers provided by the respondents and their analysis, five clusters were identified. The characteristics of the selected clusters are presented in Table 4. 

The most numerous cluster was cluster A, composed mainly of young respondents, both women and men, with a low level of education and who are a part of the labor force. Only 7.0% of the respondents were assigned to cluster B. These were mainly middle-aged women with a low level of education and not working. In cluster C (31.3%), the majority was women aged 45–59, but with secondary education and who are a part of the labor force (employed or self-employed). The least numerous was cluster D, which consisted only of women aged 35–44 and 60–75, with low or higher education and not working. The last cluster, cluster E, however, consisted only of young or older men with a low level of education and who are a part of the labor force.

### 3.4. Elements of Practice and Knowledge not Differentiating the Clusters Identified

**Figure 3:**
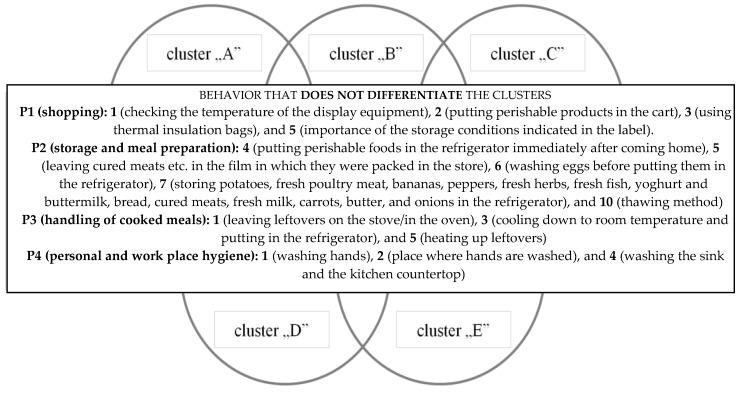
Behavior of respondents common to the clusters.

It was found that the respondents classified into particular clusters do not differ significantly in their answers to 14 out of the 25 questions about practice (Figure 3). The analysis of variance showed that all the diagnosed clusters, to an equal extent, do not pay attention to the temperature of the fridge/freezer in which food is stored in the store (P1:1) or to the order in which perishable products are placed in the cart during shopping (P1: 2) (Table 2). It was observed that all the different clusters with a similar frequency, i.e., “always” or “usually,” place food requiring storage in refrigeration temperature in a refrigerator immediately after coming home from shopping (almost 80%). Further, with a similar frequency, the clusters acted incorrectly, e.g., by leaving cured meats, cheese, and fresh meat in the film in which they were packed in the store or by washing eggs before putting them away. Persons assigned to the five clusters also similarly indicated the conditions in which they store selected products at home (P2: 7) (Figure 3). The sociodemographic characteristics that were taken into account did not influence the respondents’ practice in areas such as the time of heating a previously prepared meal before consumption and the frequency of washing hands before meal preparation.

In the second part of the questionnaire, which focused on knowledge, no differences were found between the different clusters with regard to the (K1: 1), (K2: 1). 

### 3.5. Elements of Conduct and Knowledge Characteristic (Typical) of the Identified Clusters

On the basis of an analysis of the clusters, it was found that the five clusters differed significantly in 11 out of 25 the analyzed situations (Table 5). 

Persons classified in the most numerous **cluster A** declared that they put frozen foods in their shopping cart at the end of their shopping, once they have purchased other products. At the same time, the respondents included in this cluster indicated a relatively short time of transport of frozen foods home (16 min on average). Taking into account the questions about storage and preparation of meals (P2), it can be seen that cluster A includes people who relatively rarely prepare meals at home. These people often declared that they keep UHT (Ultra High Temperature) milk and eggs in the refrigerator. They also said that they “sometimes” use the same knife (without washing) to cut raw meat and then cooked meat. It was noted that cluster A (as well as cluster E) had the lowest average value for the part of the questionnaire that pertained to knowledge (K). This indicates that the respondents classified in this group were less successful in dealing with the statements, i.e., they had less knowledge compared to the persons assigned to the other clusters, especially C and D (Table 5).

Like cluster A, **cluster B** declared the shortest time of transport of perishable products such as meat, fish, and frozen food home (13 min on average). Respondents classified into cluster B responded very similarly to those from cluster A. The LSD test indicated that in as many as 15 analyzed situations (including in the part of the questionnaire pertaining to knowledge), clusters A and B were classified as a homogeneous group (Table 6). What clearly distinguished cluster B from cluster A was less frequent placing of frozen food in the cart at the end of shopping (P1: 4), more frequent preparation of meals at home (P2: 2), and the least frequent storage of eggs in the refrigerator (Table 5). 

The persons included in **cluster C**, as often as the respondents in clusters A and E, declared that they put frozen food in the cart at the end of shopping (P1: 4). They also declared that they had learned the way they handled food daily from cookbooks and other materials published in the press (in addition to learning on their own through life experience). These persons indicated that they stored eggs at a refrigeration temperature (P2: 7) and that they washed vegetables and fruits before consumption (P2: 9). It was noted that cluster C had one of the higher average values from the knowledge part of the questionnaire (K). This indicates that the respondents classified into this group were successful in dealing with the statements, i.e., they had good knowledge compared to the persons assigned to clusters A, B, and E (Table 5).

The respondents classified into **cluster D** often gave answers that were significantly different from those given by respondents from the other clusters (Table 6). It was therefore crucial to determine in which areas and what kind of differences these were. It was found (Table 5) that the respondents place frozen food in the cart at the end of their shopping (P1: 4) the least frequently, compared to the other clusters. However, it was found that this group most often declared that they had learned how to handle food on their own through life experience and from cookbooks and other materials published in the press (as did cluster C). Persons classified into this cluster most often indicated that they prepared meals at home (P2: 2), as well as complied with the storage conditions recommended by the manufacturer (as did cluster C) (P2: 3). The average values presented in Table 5 show that this group was keen to keep all products in the refrigerator, whether or not they required it—for example, tomatoes. It was also found that this group rarely declared using the same knife to cut raw meat and then cooked meat (without washing) (P2: 8) and placing food that was still warm in the refrigerator (P3: 2). As for the issues related to personal hygiene (P4), more often than members of the other clusters, they dried their hands after washing by wiping them with a towel in the bathroom (P4: 3). Moreover, the respondents classified into this group were most successful with the statements presented in the knowledge part of the questionnaire (K), especially compared to clusters A, B, and E. (Table 5).

The last identified **cluster E** declared the least frequently that they prepared meals at home (P2: 2) and observed the storage conditions recommended by the manufacturers of products (P2: 3). In the case of this group, the lowest percentage of indications was observed for selected elements of question P2:7. Thus, these men rarely declared that they stored of UHT milk, lettuce, garlic, and tomatoes in the refrigerator. Respondents classified into group E achieved by far the worst results with regard to the statements presented in the knowledge part of the questionnaire (as did the persons in cluster A), i.e., they had much less knowledge than the persons assigned to clusters B, C, and D.

On the basis of the values presented in Table 6, it can be seen that the respondents classified into cluster A often responded similarly to those in clusters E, B, and C. For example, these four clusters declared with a similar frequency that they stored lettuce, tomatoes, and opened juice containers in a refrigerator (in all three cases, they did it less frequently than cluster D). Further, in the case of other issues raised, respondents from cluster A responded similarly (thus creating a homogenous group) to those from clusters B, C, and E, e.g., with regard to the frequency of using the same knife (without washing) to cut raw meat and then cooked meat (P2: 8), the frequency of placing meals that were still warm (leftovers) in the refrigerator (P3: 2), or the frequency of wiping hands (less frequently than cluster D) in the bathroom with a towel intended for this purpose. The respondents classified in cluster C often responded similarly to those in clusters A, D, B, and E, thus forming a homogeneous group; this was the case, respectively, 13, 11, 10, and 10 times (Table 6). Cluster of D the least frequently formed so-called homogeneous groups with other clusters, which indicates that its members most often gave definitely different answers.

## 4. Discussion

The way Polish consumers handle food and their knowledge in this area proved to be inadequate in several areas.

One of the key aspects that Polish consumers pay insufficient attention to is ensuring/maintaining the right temperature at the different stages of the technological process. As early as during shopping, the vast majority of the respondents do not pay attention to the temperature at which the purchased perishable products, e.g., raw meat or frozen products, are stored. The order in which such products are placed in the cart is also of no importance to most people participating in the study. Other authors noted similar observations, e.g., Katiyo et al. [38] found that a significant proportion of South African consumers (55%) do not follow the recommended practice of picking raw poultry meat just before leaving the store. Jevšnik et al. [6], too, observed that as much as 90% of Slovenian consumers did not pay attention to the order of purchase of raw meat during shopping and almost 70% to the temperature of the display equipment in the shop. As for the question concerning the order in which frozen food is put in the cart (P1:4), it can be concluded that the largest number of consumers put products in the cart according to the arrangement of the stands in a given store. However, as Aloysius and Binu [39] emphasize, in the standard layout of supermarkets, meat products are usually placed at the back and away from the entrance to or exit from the sales area. The above observation is of particular importance in the context of the place of shopping. In Poland [40], but also in other European countries, e.g., Spain [41], supermarkets are a very popular distribution channel for food products, among others. The proximity to home, the price, and the convenience, as well as variety and availability are important criteria in choosing these retail spaces, similar to other studies [42]. The large sales area (over 400 sq.), however, makes the shopping time significantly longer and thus the food products placed in the cart in the wrong order are subject to long exposure at room temperature. Further, the time of transport of the purchased products to homes must also be taken into account. In our own research, the average time of transport of meat, fish, and frozen food between the shop and the house was 17 min. Therefore, failure to follow the correct sequence in which food products, especially raw meat, should be put in the cart when shopping may lead to an increase in the temperature of the product and thus promote growth of any pathogenic bacteria present in the product. The use of an insulated bag or a cooling box can help to ensure that perishable foodstuffs remains at a safe temperature after purchase and during transport home [6]. In this study, a small number of the respondents (less than 14%) use heat-insulating bags to transport frozen food from the store home.

Despite the fact that the respondents did not pay much attention to the temperature of products in the shop, almost 70% of those participating in our study declared that the storage conditions recommended by the manufacturer on the label are important to them. As indicated in the studies by Wyrwa and Barska [43], for Polish consumers, shelf-life, price, ingredients, nutritional value, and content and type of preservatives are more important information on the product label than storage conditions.

Many studies have pointed out inappropriate practices and insufficient consumer knowledge about the temperature during food preparation and storage at home [12,14,44,45,46]. Meanwhile, temperature control during the technological process is one of the basic tools in controlling the growth of microorganisms in food. Failure to observe the recommended temperature values is the main cause of growth of microbial cells and, consequently, of numerous threats, including food poisoning [47] and faster food spoilage. The main reasons for the occurrence of pathogenic microorganisms in food are improper heat treatment of food (time and temperature), improper food storage conditions, including non-observance of cooling conditions during storage, and lack of care for hygiene during preparation of meals [48]. Consequently, a decision was made to focus on these aspects later in the study.

The respondents were asked how often they washed fresh eggs before putting them in the fridge. Slightly more than 40% of the respondents did this (with different frequency: sometimes/usually/always). The washing process can completely or partially damage the cuticle, which is the natural protective barrier that protects the egg from microorganisms. Therefore, such behavior can have a significant impact on the safety of the stored product but can also reduce its durability. Moreover, according to Thaivalappil et al. [49], many organizations (Centers for Disease Control and Prevention (CDC), Government of Canada, United States Department of Agriculture: Food Safety and Inspection Service) recommend not washing raw meat or eggs before preparing them to reduce the risk of foodborne illness due to cross-contamination.

In our study, less than 3/4 of the respondents declared compliance with the storage conditions of food products recommended by the manufacturers (for a similar proportion of the respondents, the storage conditions indicated by the manufacturer were important). Meanwhile, many of them stored their food incorrectly. The fact that perishable products, such as lettuce, juice in opened packaging, and fresh eggs, are relatively rarely kept in a refrigerator is particularly worrying. According to Terpstra et al. [50], although consumers have reasonably good knowledge of storage conditions, they do not always act according to this knowledge, e.g., they store vegetables incorrectly or set too high a temperature in the refrigerator. In this study, some respondents reported that they stored products in refrigerators that do not require refrigeration at all, e.g., UHT milk (almost 60% of indications). As Ruby et al. [46] pointed out, consumers often do not know that this type of milk has undergone a sterilization process (which ensures adequate product durability).

In our research, consumers’ knowledge of issues such as the temperature at which fresh poultry meat should be stored (almost 40% of correct answers), the temperature distribution inside a refrigerator (nearly 45% of correct answers) and, consequently, the place of storage in a refrigerator of, e.g., fresh meat (almost 30% of correct answers), was incomplete. Other authors also point to a lack of knowledge pertaining to these aspects. Lack of sufficient knowledge of the temperature suitable for the storage of perishable products was identified also among, e.g., Slovenian [6] and Malaysian [46] consumers. Excessively long storage of fresh poultry meat by South African consumers was reported by Katiyo et al. [38].

As shown in earlier studies [8], Polish consumers are generally aware of the fact that maintaining a cool temperature during food storage slows down the growth and development of microorganisms. However, both in earlier studies and in the study presented herein, a significant number of the respondents declared that they thawed food in the wrong way, i.e., mainly by leaving it at room temperature. Other authors also point to inappropriate knowledge and practices of consumers in this respect [6,38,44,51,52,53]. According to Roccato et al. [54], thawing, for example, of raw poultry meat overnight at room temperature caused significant increases in the number of *Salmonella Typhimurium* cases compared to thawing overnight in a refrigerator. Inappropriate knowledge and behavior with regard to frozen products are demonstrated not only by consumers but also by food production and trade professionals [55,56,57].

Knowledge of cause and effect relationships plays an important role in promoting and strengthening of proper behavior, e.g., at room temperature, microorganisms develop rapidly, and so perishable foods (fresh meat and fish) should be kept in the refrigerator and should not be thawed at room temperature. This statement has been confirmed by the results related to handling of ready-made meals. It was observed that only half of the respondents knew that leaving ready-made meals for an unlimited period of time at room temperature (on the kitchen counter) could be a health hazard. A comparable percentage of the respondents declared that they sometimes/usually/always left their leftover meals in the pot on the kitchen and/or in the oven until they were eaten.

In our study, a significant number of the respondents indicated a dangerous practice related to reheating previously prepared meals. Nearly half of them specified that they only heated up these types of meals until the dish reached a temperature that made it possible to eat it immediately. Similar behavior was observed by Jevsnik et al. [12], in whose study, 1 in 4 older Slovenians also heated up ready-made dishes until that moment, and by Lazou et al. [51] who found that 60% of Greek students did the same. The vast majority of Chinese consumers (over 70%) are aware of the fact that previously prepared meals should be very carefully heated before consumption [53].

It was found that the vast majority of the respondents handled raw fruit and vegetables properly before eating them, i.e., washed them. It was also found that the respondents had appropriate knowledge in this respect, i.e., they were aware that this process reduces the number of microorganisms present on the surface of the products. Other authors, in other countries, also did not report problems in this area, e.g., in China [53], Palestine [58], Slovenia [6], and India [59]. However, Lazou et al. [51] and Vlasin-Marty et al. [11] found that the vast majority of their respondents, i.e., Greeks (over 70%) and Americans (85%), carried out the process of washing of fruits and vegetables in the correct way, in cold running water. Thus, our own results and those presented by other authors lead to the conclusion that awareness of the presence of biological contaminants [60] but also physical and chemical contaminants [61] on the surface of products, as well as the teaching of the “wash fruits before eating” principle since childhood, has a positive impact on this behavior.

While the respondents were less successful with questions related to knowledge of food heat treatment and storage parameters, it can be observed that they more often provided correct answers to questions related to personal hygiene or workplace, which has also been reported by other authors [46]. The vast majority of the respondents declared that they washed their hands before preparing a meal. In other studies [2,59], too, respondents usually declared that they did so. Hand hygiene is considered to be the basic measure limiting the spread of infectious diseases [62]. Adequate hand hygiene is an important way to eliminate pathogens present on the surface of hands and thus is an effective way to combat infections that often require home treatment and often hospitalization. Judah et al. [63] and Randle et al. [64] emphasize that approximately one-third of infections can be prevented by improving hand washing practices.

We also observed that the respondents maintain cleanliness in the kitchen. A small percentage of the respondents indicated that they washed sinks and countertops less frequently than once a day. This simple activity is extremely important, especially in the case of work surfaces in the kitchen, because there are, e.g., fecal flora microorganisms and mold fungi, which find humid environment to be particularly favorable, on many surfaces used daily [65]. Relatively frequent cleaning of work surfaces in the kitchen was also indicated by Bablu et al. [59], Awang Teh et al. [66], and Sanlier [2].

In this study, in addition to learning about the food safety knowledge and practices of Polish consumers, their segmentation was also made in order to identify groups characterized by similar ways in which they handle food. In our research, five segments of consumers were identified who differ in their behavior and knowledge, taking into account sociodemographic characteristics such as gender, age, education, and professional activity.

Drawing conclusions on the basis of the cluster analysis that was conducted and the five clusters identified on its basis proved to be a difficult task. This is because, in many cases, these clusters formed so-called homogeneous groups. However, some basic characteristics were observed that differentiate the respondents taking part in the survey.

It was found that cluster D was clearly distinct from the other four. The cluster was made up of women over 35 years of age, with different education, who were not a part of the labor force (unemployed, housewives, and pensioners). According to their declarations, they were the ones who most often prepared meals at home and they learned the food handling methods they currently used, more often than the persons in the other clusters, through their own life experience and by using cookbooks and other materials published in the press. The women from this cluster most often gave correct answers to many of the questions included in the questionnaire, e.g., observing the storage conditions recommended by the manufacturer, storing open juice containers in the refrigerator, very rare use of the same knife to cut raw meat and then cooked meat, and wiping hands after washing with a towel in the bathroom. They also showed the best knowledge of food safety. Further, correct answers, forming homogenous groups with segment D, were frequently given by people in cluster C, also mainly women over 35 years old, but mostly with secondary education and a part of the labor force. The most frequent incorrect behaviors, but also the most limited knowledge in the area in questions, were identified in the case of cluster E (young men with low education and active in the labor force) and cluster A (young women with low education and active in the labor force).

Therefore, it was found that the respondents’ food safety knowledge translates into correct behavior. This relationship is not always confirmed by other studies. Byrd-Bredbenner et al. [13], Ergönül [45], and Hassan and Dimassi [67], among others, demonstrated that a good level of knowledge does not always correspond to proper behavior. As Sharif and Al-Malki [68] and Woh et al. [69] pointed out, it may also be the case that the respondents act correctly, despite the lack of sufficient knowledge in a given area. This may be due to the fact that the respondents learn certain practices in their homes or from observation and perform them without considering their validity [8].

As in earlier studies [8,70,71,72,73,74], it was found that it was women (clusters D, C, and B) who demonstrated better food safety knowledge and practices than men (clusters E and A). On the basis of the characteristics of the identified clusters, it can be seen that this is one of the key factors determining the correctness of the answers given. One should keep in mind that, in many countries, women still are responsible for preparing meals for the family. That is why they are more aware of the things they do every day in the kitchen. However, it should also be taken into account that in many countries, including Poland, women are increasingly involved in professional work and men are increasingly involved in daily household duties. Therefore, the results of our research indicate a strong need to educate men in food safety.

Another factor determining the correctness of the answers given by the respondents, although it seems to be much less important than gender, is the age of the respondents. Clusters E and A, i.e., groups that showed much less knowledge and practices in the area of food safety, were composed of a larger percentage of young people (up to 35 years of age), compared to other clusters, i.e., approx. 36%–38%. Some authors have pointed out that the level of food safety knowledge and behavior increases with the age of respondents [75]. Elderly consumers can use their life experience in everyday life while preparing meals [76].

The cluster analysis that was conducted made it possible to create a comprehensive picture of consumers in terms of sociodemographic features. It can be concluded that consumers who have adequate knowledge of food safety and make appropriate use of it (cluster D) are women who, due to their low professional activity, often prepare meals. They also have some life experience (people over 35 years of age), which they undoubtedly use in their work at home. Education plays a lesser role, as this group includes women with both elementary and higher education.

On the other hand, clusters E and A (less food safety knowledge and practices) are composed mostly of men of different ages, with a clear prevalence of persons with elementary or secondary education and a part of the labor force. They have less time to prepare meals, and so they pay less attention to the activities performed in the kitchen. It can also be noted that the respondents classified into these two clusters, more often than those in cluster D, indicated the correct order of placing frozen food in the shopping cart. This may indicate that, due to lack of time, they more often buy ready-made dishes, including those in frozen form, and so they know how to handle them.

The consequence of improper handling of food at the stage of purchase, storage, and preparation of meals, apart from health problems, can be food waste. One of the basic reasons for throwing food away in Polish households is its spoilage [33]. The activities that were observed that can definitely reduce the shelf life of products/meals are washing eggs before they are placed in the fridge, inappropriate storage of products, e.g., juices in open packaging, inappropriate thawing of products, and keeping cooked food at room temperature for too long. This is a sequence of incorrect operations that can affect the durability of prepared meals and thus the level of food waste in households. Of course, the results presented in this paper provide a certain basis for continuing research in this direction.

Consumers have a negative attitude towards food waste [77,78]. This is because they have, above all, the feeling that, together with the food, they throw away money [18]. Therefore, paying more attention to the multidimensional nature of improper food handling and pointing out that it can not only have an adverse effect on health but also can create an additional financial burden are a good basis for promoting appropriate food-related practices among consumers.

### Limitations and Further Research

That other features such as family income and living place were not taken under consideration is a limitation of this study. Including these features would give a full description of the different groups of respondents. Future studies should consider other sociodemographic factors not included in this segmentation, e.g., living place, number of people in household including children, family income and other factors related to lifestyle.

## 5. Conclusions

This paper identifies incorrect behavior and some lack of knowledge of Polish consumers concerning food safety issues. It was found that a significant number of the respondents did not pay attention to the order in which they put perishable products and frozen food in the cart during shopping, did not properly store food products at home (e.g., juices in open packaging), did not properly thaw frozen food, kept cooked meals at room temperature for too long, or heated up leftover dishes for too short a time.

Our study allowed us to identify five segments made up of consumers who differed in their behavior and knowledge of food safety issues, including sociodemographic characteristics such as gender, age, education, and professional activity. The segment that was characterized by the best conduct and knowledge of food safety issues consisted exclusively of unemployed women over 35 years of age. At the same time, this segment was the least numerous of the five identified. On the other hand, the least knowledge and practices were demonstrated by two segments consisting mainly of men of different ages, with a clear prevalence of people with primary and secondary education and a part of the labor force. Unfortunately, these two segments constituted a majority of the studied population. 

The segmentation provided valuable information which indicates that educational programs on food safety need to be further strengthened. These programs should be aimed primarily at young consumers who are a part of the labor force. Consumers’ attention should be drawn not only to the health aspects, but also to the economic context of food waste.

## Figures and Tables

**Table 1:** Sociodemographic characteristics of the respondents (*n* = 1115).

Feature	Group	Number of Respondents (*n*)	Percentage (%)
Gender	women men	570 545	51.1 48.9
Age	18–34 y.o. 35–44 y.o. 45–59 y.o. more than 60 y.o.	314 208 304 289	28.2 18.6 27.3 25.9
Education	elementary, vocational secondary higher	450 468 197	40.3 42.0 17.7
Labor force participation	employed or self-employed others (students, unemployed, housewife; pensioner/retiree, farmer)	720 395	64.6 35.4

**Table 2:** Respondent’s food handling practices during shopping and at home (P).

No.	Answers	%
P1: 1	**(a) always/usually**	**10.7**
	(b) sometimes/rarely/never	89.3
P1: 2	**(a) always/usually**	**19.1**
	(b) sometimes/rarely/never	80.9
P1: 3	**(a) always/usually**	**13.6**
	(b) sometimes/rarely/never	86.6
P1: 4	(a) at the start of shopping	2.8
	(b) I put in the products according to the arrangement of the stands in the store	49.1
	**(c) at the end of the shopping, once other products have been purchased**	37.6
	(d) I do not shop in self-service stores	2.3
	(e) I do not buy frozen foods	8.2
P1: 5	**(a) definitely relevant/rather relevant**	67.9
	(b) neither relevant, nor irrelevant/rather irrelevant/definitely irrelevant	32.1
P1: 6	(a) up to 10 min.	38.5
	(b) 10-20 min.	40.0
	(c) 21-30 min.	16.9
	(d) more than 30 min.	4.6
P2: 1	(a) by myself (life experience)	52.7
	(b) at school	5.8
	(c) from cookbooks/other materials published in the press	6.0
	(d) from TV/Internet programs	9.8
	(e) from a life partner	16.1
	(f) from parents	62.7
	(g) cooking is my profession	2.0
	(h) in the military	0.09
P2: 2	**(a) always/usually**	54.1
	(b) sometimes/rarely/never	45.9
P2: 3	**(a) always/usually**	66.8
	(b) sometimes/rarely/never	33.2
P2: 4	**(a) always/usually**	78.9
	(b) sometimes/rarely/never	21.1
P2: 5	**(a) never/rarely**	32.1
	(b) sometimes/usually/always	67.9
P2: 6	**(a) never/rarely**	57.1
	(b) sometimes/usually/always	42.9
P2: 7	1: UHT (Ultra High Temperature) milk; **2:** lettuce; **3:** potatoes; **4:** fresh poultry; **5:** garlic; **6:** bananas; **7:** peppers; **8:** fresh herbs; **9:** fresh fish; **10:** yoghurt, buttermilk, etc.; **11:** bread; **12:** cured meats; **13:** fresh milk; **14:** carrots; **15:** butter; **16:** tomatoes; **17:** eggs; **18:** onion; **19:** opened juice containers;	**1:**58.5; **2:**48.1; **3:**2.6; **4:**81.5; **5:**13.4; **6:**11.0; **7:**30.8; **8:**9.6; **9:**76.6; **10:**91.0; **11:**3.7; **12:**91.5; **13:**81.0; **14:**27.1; **15:**84.0; **16:**35.5; **17:** 78.5; **18:**16.7; **19:**63.3
P2: 8	**(a) never/rarely**	51.7
	(b) sometimes/usually/always	48.3
P2: 9	**(a) always/usually**	84.3
	(b) sometimes/rarely/never	15.7
P2: 10	**(a) I put it in the fridge**	17.0
	**(b) in a microwave oven**	9.1
	(c) I immerse it in warm water	23.9
	(d) I leave it on the kitchen counter until it thaws	45.3
	(e) I do not use frozen foods	4.8
P3: 1	**(a) never/rarely**	54.1
	(b) sometimes/usually/always	45.9
P3:2	**(a) never/rarely**	81.8
	(b) sometimes/usually/always	18.2
P3: 3	**(a) always/usually**	49.6
	(b) sometimes/rarely/never	50.4
P3: 4	**(a) always/usually**	15.5
	(b) sometimes/rarely/never	84.5
P3: 5	(a) I do not reheat such a meal	2.7
	(b) to a temperature that enables it to be eaten immediately, i.e., it is warm but not too hot	48.9
	(c) until the meal is boiling	31.2
	**(d) until it has boiled for a few minutes**	13.8
	(e) not applicable	3.4
P4: 1	**(a) always/usually**	85.0
	(b) sometimes/rarely/never	15.0
P4: 2	(a) in the kitchen sink	67.7
	(b) in the bathroom	32.3
P4: 3	(a) I don’t dry my hands; they get dry by themselves	4.0
	(b) wipe them with the kitchen apron and/or other clothing	5.8
	(c) with a cloth for wiping clean dishes	16.2
	**(d) with disposable paper towels**	26,2
	**(e) with a cloth for wiping hands in the kitchen**	28.2
	**(f) with a towel for wiping hands in the bathroom**	19.6
P4: 4	(a) after each use	23.0
	(b) after each main meal, such as breakfast, lunch, and dinner	23.9
	(c) when I see they are dirty	35.8
	(d) once a day	9.5
	(e) less frequently than once a day	7.8

* The correct answers are marked with the bold fonts.

**Table 3:** Respondents’ food safety knowledge (K).

No.	Answers *	%
K1: 1	(a) the lightest products first and the heaviest products at the end, so as not to get tired by pushing the cart	9.7
	**(b) first non-food products, followed by food products that do not require storage in a refrigerator, and finally fresh food that requires storage in low temperatures**	25.7
	(c) first products placed the closest to the entrance, then according to the arrangement of the shelves towards the cash registers	25.6
	(d) the order in which the products are placed in the cart during shopping is of no importance	38.9
K1: 2	**(a) −2 °C to +4 °C**	38.1
	(b) above +4 °C to +6 °C	34.9
	(c) above +6 °C to +10 °C	3.1
	(d) I don’t know, it’s hard to tell	23.8
K2: 1	(a) on the top shelf	18,1
	(b) on the middle shelf	19.7
	**(c) on the bottom shelf**	29.4
	(d) the height of the shelf is irrelevant	18.6
	(e) I don’t know, it’s hard to tell	14.2
K2: 2	**(a) I definitely don’t agree/I rather disagree**	44.8
	(b) I neither agree nor disagree/I rather agree/I strongly agree	55.2
K2: 3	**(a) I strongly disagree/I rather disagree**	47.8
	(b) I neither agree nor disagree/I rather agree/I strongly agree	52.2
K2: 4	**(a) I strongly disagree/I rather disagree**	42.3
	(b) I neither agree nor disagree/I rather agree/I strongly agree	57.7
K2: 5	**(a) I strongly agree/I rather agree**	75.4
	(b) I neither agree nor disagree/I rather disagree/I strongly disagree	24.6
K2: 6	**(a) I strongly agree/I rather agree**	68.8
	(b) I neither agree nor disagree/I rather disagree/I strongly disagree	31.2
K2: 7	**(a) I strongly disagree/I rather disagree**	38.2
	(b) I neither agree nor disagree/I rather agree/I strongly agree	61.8
K2: 8	**(a) I strongly disagree/I rather disagree**	74.2
	(b) I neither agree nor disagree/I rather agree/I strongly agree	25.8
K3: 1	**(a) I strongly disagree/I rather disagree**	50.3
	(b) I neither agree nor disagree/I rather agree/I strongly agree	49.7
K3: 2	**(a) I strongly disagree/I rather disagree**	20.2
	(b) I neither agree nor disagree/I rather agree/I strongly agree	79.8
K4: 1	**(a) I strongly agree/I rather agree**	74.6
	(b) I neither agree nor disagree/I rather disagree/I strongly disagree	25.4
K4: 2	**(a) I strongly disagree/I rather disagree**	53.0
	(b) I neither agree nor disagree/I rather agree/I strongly agree	47.0

* The correct answers are marked with the bold fonts.

**Table 4:** Characteristics of the clusters.

Cluster	N	% Share of the Cluster in the Studied Population	Share in the Cluster (%)			
			Sex ^(a)^	Age ^(b)^	Education ^(c)^	Labor Force Participation ^(d)^
**A**	**380**	34.1	F:47.9 **M:52.1**	**1:36.1** 2:28.9 3:12.4 4:22.6	**P:51.1** S:44.5 H:4.5	**W:69.2** O:30.8
**B**	78	7.0	**F:69.2** M:30.8	1:0 2:0 **3:100** 4:0	**P:79.5** S:20.5 H:0	W:30.8 **O:69.2**
**C**	349	31.3	**F:82.2** M:17.8	1:18.9 2:25.2 **3:29.2** 4:26.6	P:4.6 **S:60.5** H:35.0	**W:70.5** O:29.5
**D**	16	1.4	**F:100** M:0	1:0 **2:50** 3:0 4:50	**P:50** S:0 H:50	W:0 **O:100**
**E**	292	26,2	F:0 **M:100**	**1:38.4** 2:0 3:27.7 4:33.9	**P:57.5** S:23.6 H:18.8	**W:63.7** O:36.3

^(a)^ F: female; M: male; ^(b)^ 1: 18–34 y.o.; 2: 35–44 y.o.; 3: 45–69 y.o.; 4: 60–75 y.o.; ^(c)^ P: elementary; S: secondary; H: higher; ^(d)^ W: employed or self-employed; O: others (students, unemployed, housewife; pensioner/retiree, farmer).

**Table 5:** Average level of frequency rate * or arithmetic mean ** for the identified clusters with the results of the variance analysis and the least significant difference (LSD) test.

Question ^(II)^		Cluster ^(I)^					*p*-Value
		A	B	C	D	E	
P1: 4 *	a.	0.05	0.02	0.04	0.00	0.01	-
	b.	0.35	0.33	0.33	0.43	0.33	0.913
	c.	0.46 ^c^	0.31 ^b^	0.47 ^c^	0.21 ^a^	0.40 ^bc^	0.023
P1:6 ** (transport time: min.)		15.6 ^ab^	12.7 ^a^	17.8 ^bc^	19.3 ^c^	18.6 ^c^	0.000
P2: 1 *	a.	0.47 ^a^	0.58 ^a^	0.59 ^a^	0.79 ^b^	0.45^a^	0.020
	b.	0.07	0.04	0.07	0.00	0.05	0.422
	c.	0.05 ^a^	0.05 ^a^	0.10 ^b^	0.14^b^	0.02 ^a^	0.025
	d.	0.08	0.07	0.14	0.14	0.09	0.478
	e.	0.12	0.13	0.11	0.00	0.28	0.086
	f.	0.69	0.59	0.63	0.64	0.63	0.799
	g.	0.00	0.05	0.04	0.07	0.00	0.090
P2: 2 **		2.62 ^c^	1.81 ^b^	1.98 ^b^	1.36 ^a^	3.19 ^c^	0.002
P2: 3 **		2.23 ^bc^	2.15 ^b^	2.01 ^ab^	1.79 ^a^	2.36 ^c^	0.003
P2: 7 *	1. UHT milk	0.64 ^b^	0.59 ^ab^	0.62 ^b^	0.57 ^ab^	0.48 ^a^	0.021
	2. lettuce	0.47 ^a^	0.41 ^a^	0.54^ab^	0.71 ^b^	0.41 ^a^	0.007
	5. garlic	0.12 ^a^	0.10 ^a^	0.20 ^b^	0.21 ^b^	0.10 ^a^	0.049
	16. tomatoes	0.36 ^a^	0.29 ^a^	0.39 ^a^	0.57 ^b^	0.30 ^a^	0.031
	17. eggs	0.80 ^b^	0.65 ^a^	0.84 ^b^	0.71 ^ab^	0.77 ^b^	0.023
	19. opened juice containers	0.63 ^a^	0.53 ^a^	0.67 ^ab^	0.79 ^b^	0.62 ^a^	0.032
P2: 8 **		3.41 ^a^	3.66 ^a^	3.79 ^a^	4.64 ^b^	3.58 ^a^	0.000
P2: 9 **		1.61 ^b^	1.64 ^b^	1.49 ^a^	1.43 ^a^	1.86 ^b^	0.021
P3: 2 **		4.47 ^a^	4.64 ^a^	4.50 ^a^	5.00 ^b^	4.38 ^a^	0.043
P3: 4 **		3.55 ^ab^	3.74 ^b^	3.32 ^a^	3.50 ^ab^	3.73 ^b^	0.031
P4: 3 *	a.	0.02	0.01	0.03	0.07	0.06	0.178
	b.	0.03	0.06	0.06	0.00	0.06	0.368
	c.	0.16	0.15	0.13	0.14	0.19	0.695
	d.	0.33	0.29	0.30	0.21	0.25	0.520
	e.	0.26	0.28	0.31	0.07	0.24	0.174
	f.	0.20 ^a^	0.21 ^a^	0.18 ^a^	0.50 ^b^	0.21 ^a^	0.031
K2:2–K2:8 and K4 and K4 **		37.2 ^ab^	38.8 ^b^	39.4 ^bc^	42.8 ^c^	36.5 ^a^	0.000

^(I)^ An identical letter at the arithmetic mean value or frequency rate means that there are no significant differences between the clusters. ^(II)^ Direction of the scale: **practice (frequency)**: from 1—“always” to 5—“never”; **knowledge:** from **1** “I strongly agree” to **5** “I strongly disagree,” except for statements K2:5, K2:6, and K4:1, where 1—“I strongly disagree” to **5**—“I strongly agree”.

**Table 6:** A matrix showing the scale of similarity between the clusters (the cells show the number of cases in which a given cluster formed a homogenous group with other clusters—LSD test).

	Clusters					
**Clusters**		**A**	**B**	**C**	**D**	**E**
	**A**	x	15	13	3	16
	**B**	15	x	10	3	13
	**C**	13	10	x	11	10
	**D**	3	3	11	x	4
	**E**	16	13	10	4	x
